# Pharmaceutical and pharmacological studies of Shen Ma Yi Zhi granule for prevention of vascular dementia: A review

**DOI:** 10.3389/fnins.2022.1044572

**Published:** 2022-11-24

**Authors:** Su-rui Chang, Jian-gang Liu, Hao Li, Mei-xia Liu, Dan-dan Shi, Li-juan Zhou

**Affiliations:** ^1^Xiyuan Hospital, China Academy of Chinese Medical Sciences, Beijing, China; ^2^Graduate School, China Academy of Chinese Medical Sciences, Beijing, China; ^3^National Clinical Research Center for Chinese Medicine Cardiology, Beijing, China; ^4^Institute of Geriatrics of China Academy of Chinese Medical Sciences, Beijing, China; ^5^Wangjing Hospital, China Academy of Chinese Medical Sciences, Beijing, China

**Keywords:** Shen Ma Yi Zhi, VaD, traditional compounds, animal models, signal pathway

## Abstract

**Background:** With dementia significantly increasing hospitalization and disability rates, worldwide aging of the population presents major challenges to public health. The majority of cases of cognitive dysfunction among the elderly, however, are characterized by an identifiable, preventable and treatable vascular component. As such, increased study of preventative methods in the context of dementia is warranted. Traditional Chinese medicine compounds have been reported to be neuroprotective and improve cognitive function via a variety of mechanisms. Shen Ma Yi Zhi granule (SMYZG) is one such collection of compounds that has been proven clinically effective. Pharmacological mechanisms of action, pharmacokinetics and clinical applications of SMYZG have been previously studied using a variety of vascular dementia animal models. SMYZG activates and regulates four main signaling pathways relevant to vascular dementia including the AMPK/PPARα/PGC-1α/UCP2, Nrf2/HO-1, HIF-1/VEGF/Notch, and VEGF/Flk-1/p8 MAPK pathways. Furthermore, SMYZG influences anti-inflammatory and anti-oxidant stress responses, reverses demyelination of brain white matter and vascular endothelium, regulates pericyte function and normalizes mitochondrial metabolism. Neuroprotective effects of SMYZG, as well as those promoting regeneration of vascular endothelium, have also been reported in studies of rat models of vascular dementia. Future research concerning SMYG is warranted for development of vascular dementia preventative management strategies.

## Introduction

Cognitive impairment significantly impairs thought, communication, comprehension, and memory formation processes. Vascular risk factors are among the leading etiologies of cognitive impairment among the elderly. Therapies capable of effectively delaying, preventing or treating cognitive decline, however, remain to be developed for clinical use. Recently, a number of studies have reported success in treating Alzheimer’s disease and vascular dementia with traditional Chinese medicine compounds as demonstrated using behavioral tests, histopathological examinations and indexes relevant to neurotransmitter catabolism. Importantly, these studies underscored the excellent potential that traditional Chinese medicine compounds have in future clinical use (Lecordier et al., 2021).

Shen Ma Yi Zhi granule (SMYZG), a Chinese herbal prescription, was demonstrated effective in treating vascular disease (Chang Surui, 2020). This compound consists of ginseng (*Panax Ginseng C.A. Mey*), Gastrodia elata (*Gastrodia elata Bl*), Euonymus alatus [*Euonymus alatus (Thunb.) Sieb*], and Ligustici (*Ligusticum chuanxiong Hort*). Pharmaceutical, pharmacodynamic and toxicological have been finished to determine the pharmaceutical extractions routing. *In vivo* studies have similarly been conducted (Li, 2017; Kun et al., 2020; Sun et al., 2021a,b; Lijuan et al., 2022). Importantly, aqueous extracts of SMYZG were reported to not only be neuroprotective but also beneficially affect learning and memory, hippocampal structure, central cholinergic system function as well as suppress the inflammatory and oxidative stress responses based on 2-VO and MID models; the primary active functional components were reported to be ginsenosides, gastrodin, ferulic acid, and quercetin (Nan-Nan et al., 2019). Toxicological studies revealed no negative effects on organs or critical biological parameters. Furthermore, SMYZG was awarded a National Invention Patent (Meixia, 2020) and recorded in the national registry of Chinese medicine preparations (No. Z20200005000).

## Chemistry of Shen Ma Yi Zhi granule

### Preparation processes

**FIGURE 1:**
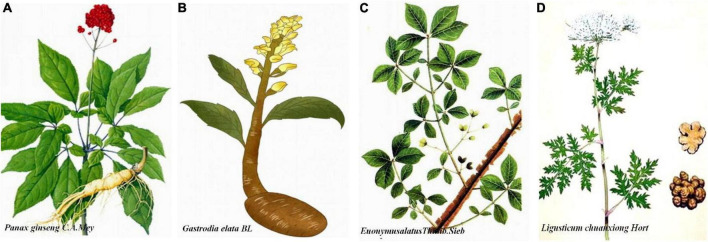
Detailing of herbal Shen Ma Yi Zhi granule (SMYZG) components. **(A)** *Panax Ginseng C.A. Mey*; **(B)** *Gastrodia elata Bl*; **(C)** *Euonymus alatus (Thunb.) Sieb*; **(D)** *Ligusticum chuanxiong Hort*. Sources for the images within this figure: 699pic.com and Chinese Pharmacopoeia.

Shen Ma Yi Zhi granule is composed of ginseng (*Panax Ginseng C.A. Mey*), Gastrodia elata (*Gastrodiaelata Bl*), Euonymus alatus [*Euonymus alatus (Thunb.) Sieb*], and Ligustici (*Ligusticum chuanxiong Hort*) in a respective 3:3:3:2 ratio. All components meet the standards set forth by the Chinese Pharmacopoeia Commission (2015). Constituent weighting is achieved using the analytic hierarchy process (AHP) method. Orthogonal and single factor analyses were applied for optimization of extraction and purification technology used to prepare SMYZG by measuring yield rates of extraction and transition probability of gastrodin, *p*-hydroxybenzylalcohol, and ferulic acid. Briefly, Gastrodia elata, Euonymus alatus, and Ligustici slices are mixed, with water added to the mixture three times for 1 h each time. Ten times the amount of water per compound is added the first time while eight times the amount of water is added the second and third times. Ginseng slices are soaked in water for compound extraction twice and at 2 h per time; 12 times the amount of water is added to the ginseng the first time and 10 times the second. Mixing the extracts together in the Drug Manufacturing Room of Xiyuan Hospital, Chinese Academy of Chinese Medical Sciences, produces a crude drug extract of 2.44 g (Figure 1).

### Chemical components of Shen Ma Yi Zhi granule

Ginseng (*Panax Ginseng C.A. Mey*), a root of Panax (Araliaceae), contains panaxosides A, B, C, D, E, and F, volatile oil, Ginseng ene, vitamin B1, vitamin B2, nicotinic acid, niacinamide, pantothenic acid, choline, maltase, invertase, esterase, and a variety of amino acids (Jian and Shao-wa, 2021). Gastrodia elata (*Gastrodiaelata Bl*), i.e., dried Gastrodia elata plant content, contains compounds such as Gastrodia elata glucoside and Gastrodia elata ether glucoside (Wei et al., 2021). Euonymus alatus [*Euonymus alatus (Thunb.) Sieb*], the twig outgrowths of celastraceae plants, contains compounds such as stigmast-4-en-3-one, quercetin, β-sitosterol, dehydrodicatechin, aromadendrin, d-catechin, 4 β-sitosterone, alatamine, and wilfordine (Lei et al., 2015; Rui-xi et al., 2015; Yanxiu et al., 2021). Ligustici (*Ligusticum chuanxiong Hort*), i.e., dried Ligusticum plant content, contains compounds such as tetramethylpyrazine, perlolyrine, ligustilide, wallichilide, senkyunolide, vanillic acid, caffeic acid, protocatechuic acid, and ferulic acid (Jiangang et al., 2019; Li Qian, 2020; Zhonghui et al., 2020).

**FIGURE 2:**
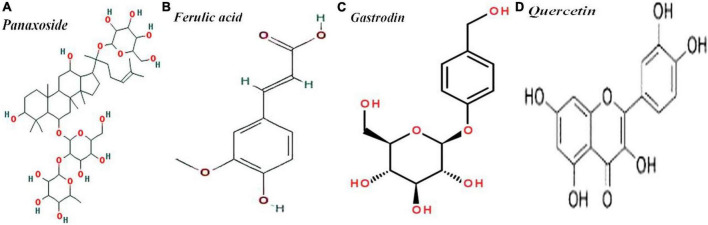
Molecular structures of **(A)** panaxoside/C36H62O8, **(B)** ferulic acid/C10H10O4, **(C)** gastrodin/C13H18O7, and **(D)** Quercetin/C15H10O7.

Importantly, there are four major clinically effective components of SMYZG, including *b*-D-glucopyranoside (3b, 12b)-3,12-dihydroxydammar-24-en-20-yl, 4 -(hydroxymethyl)phenyl-β-D-glucopyranoside, 4H-1-benzopyran-4-one, 2-(3,4-dihydroxyphenyl)-3,5,7-trihydroxy-flavone, 2–propenoic acid and 3-(4-hydroxy-3-methoxyphenyl). Molecular structures are detailed in Figure 2.

**FIGURE 3:**
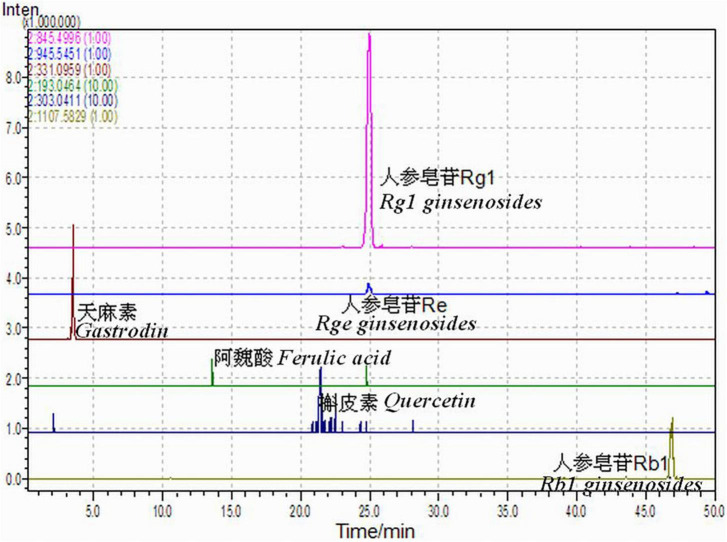
Shen Ma Yi Zhi granule (SMYZG) sample chromatogram absorption peaks and HPLC retention times.

High-performance liquid chromatography parameters of SMYZG, shown in Figure 3, are as follows: chromatographic column, Tnature C18 column (4.6 mm × 250 mm, 5 min); drug elution times: 0–5 min, 0–14%; 6–10 min, 14–19%; 11–15 min, 19–20%; 16–20 min, 20–24%; 21–25 min, 20–24%. Mobile phase: methanol (A) approximately 0.1%; acetum (B) approximately 0.1%. Flow rate: 0.25 ml/min. UV detection wavelength: 321 nm. Injection volume: 5 μl (Figure 3).

## Clinical studies concerning Shen Ma Yi Zhi granule

**FIGURE 4:**
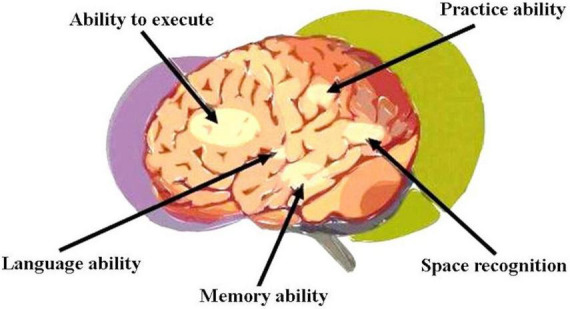
Functional regions (vascular control areas) involved in cortical dementia.

Vascular dementia (VaD), the second commonest form of dementia after Alzheimer’s disease (Huang et al., 2016), is primarily characterized by poor athletic performance, executive functioning, information processing, concentration, and memory (O’Brien et al., 2003; Hachinski et al., 2006; Moorhouse and Rockwood, 2008). Diagnosis is not always straightforward as a number of non-specific signs and wide variety of risk factors are often present among older patients. In contrast to Alzheimer’s, VaD generally manifests more acutely with executive functioning gradually declining and memory impairment fluctuating (Hachinski et al., 2006; Moorhouse and Rockwood, 2008). Functional regions (vascular control areas) involved in cortical dementia are shown in Figure 4.

Wu et al. (2017) recruited 60 mild and moderate VaD patients previously diagnosed according to Chinese guidelines for diagnosis and treatment of vascular cognitive impairment (Cognitive Impairment Committee NBCM, 2019), then observed the hemorheological indexes and tested their cognitive functioning. Mini-Mental State Exam (MMSE) scores of patients treated with SMYZG were found to have markedly improved; as such, SMYZG treatment was concluded to effectively improve cognition among this patient population. And it increased erythrocyte deformability, inhibited platelet function, reduced blood viscosity and improved the blood rheology.

One randomized controlled trial (Zhang et al., 2020) evaluating the clinical effectiveness of treating VaD patients with SMYZG in combination with Ginkgo biloba tablets revealed that this regimen increases MMSE scores, decreases Alzheimer’s Disease Assessment Scale-Cognitive Subscale (ADAS-Cog) scores, improves patient memory, attenuates mood fluctuations and improves ADL capacities. Furthermore, these compounds were noted to increase blood levels of both nitric oxide (NO) and vascular endothelial growth factor (VEGF) and thus improve endothelial function. In experimental group, the level of neuronspecific enolase (NSE) decreased and the level of brain-derived neurotrophic factor (BNDF) increased indicated that SMYZ had a certain clinical effect in nourishing nerves and repairing damaged nerves.

## Model animal experiments

Selection of suitable animal models is key in successful experimental design and is also critical to result accuracy and objectivity. At least 10 methods of creating VaD animal models have been reported and include procedures such as vessel occlusion (VO), lacunar infarct embolization (MID) and photochemical induction (Lin et al., 2014). Studying multiple VaD animal models is therefore helpful in evaluating pharmacological mechanisms more precisely.

### 2-VO rat model data

The 2-VO rat model leads to chronic cerebral hypoperfusion and subsequent development of secondary cerebrovascular pathophysiological alterations, critical risk factors for VaD pathogenesis (Zhang et al., 2018). Advantages of this model include lower levels of injury inflicted on animals and rapid operating time. This model simulates hypoperfused (Tomimoto et al., 2003; Farkas et al., 2007) and hypoxic (Hu et al., 2017) states of the human brain, especially in structures related to recognition such as the hippocampus and cerebral cortex. These brain regions are more easily affected by oxidative stress, with resultant structural neuronal and cholinergic system damage significantly impairing learning, memory, and behavior (Luo et al., 2019; Zhu et al., 2019; Fan et al., 2020). Relevant mechanisms of these pathological processes are closely related with those of chronic cerebral ischemia (CCI) (Cheng et al., 2019).

Frequency of Morris water maze platform searching, frequency of original platform crossing and percentage of time spent swimming in the quadrant of the original platform location among rats in SMYZG treatment groups increased significantly. These data underscore that SMYZG improves learning and memory function among 2-VO rats. Importantly, SMYZG repairs 2-VO rat cortical damage by improving the loose arrangement of pyramidal cells, reducing axonotmesis and neuronal shrinkage, protecting the morphology and structure of neuronal mitochondria, as well as increasing the number of neuronal mitochondrial and surrounding microvasculature density.

### MID rat model data

Multiple cerebral infarction, also known as white matter disease, is most commonly caused by systemic disease or brain lesions (Kalaria, 2016). Cerebral small vessel disease (CSVD) is related to the occurrence and development of MID and is one of the most common causes of VaD. MID patients with cognitive impairment frequently exhibit significant small vessel ischemic changes along with areas of cerebral infarction (Jiang et al., 2021). Many studies have utilized sludged blood or kelp microgelation (KMG) to construct MID rat models (Qun and Yongjun, 2015). Successful model construction is demonstrated by confirmation of multiple deep lacunar infarctions. Model rats exhibit obvious post-operative cognitive disorders (Qun and Yongjun, 2015).

Zhou et al. (Hao, 2020) utilized KMG for MID rat model construction. Among SMYZG treatment group rats, escape latency and swim distance in the platform quadrant were significantly shortened; the frequency of original platform crossing and percentage of swimming time, as well as percentage of swimming distance in the original platform quadrant were all significantly increased. Wen et al. reported that SMYZG ameliorates neuronal pathogenesis, shortens distances between pyramidal cells, increases Nissl body quantities, attenuates mitochondrial dysfunction and improves endoplasmic reticular function in the MID rat model (Li, 2017).

### APOE^–/–^ mice data

The Apolipoprotein E knock-out (ApoE^–/–^) mouse model is widely used to study atherosclerosis. ApoE^–/–^ mice possess risk factors such as cerebral arteriosclerosis and lipometabolic disturbances. Blood-brain barrier disruptions and synaptic injury are among the pathological characteristics seen in this mouse model, as in VaD. Masliah et al. (1997) reported that 3-month-old ApoE^–/–^ mice exhibit poorer learning and memory functionality as compared to control mice. Other studies similarly reported ApoE^–/–^ mice to manifest neuropathologic changes resulting in worsened memory formation, among others. Along with worsened defense against oxidative damage in the ApoE^–/–^ mouse brain, antioxidant enzyme levels decrease (Shea et al., 2002) and the hippocampus is significantly affected (Charles et al., 2000).

Screening of proteins expressed in vascular endothelium involved in brain lacunar infarcts (Wang et al., 2018) revealed pathologic changes in cerebral vascular endothelial cell permeability and vasodilation among ApoE^–/–^ mice. Proteomics and network pharmacology suggests that SMYZG improves cognition via regulation of eNOS and CAV1 expression (Qiong et al., 2019).

## Mechanistic studies concerning prevention of vascular dementia by Shen Ma Yi Zhi granule

Vascular dementia is commonly caused by hypoxic-ischemic brain damage via a number of mechanisms. Multiple infarcts can occur in the setting of numerous cardiovascular and cerebrovascular diseases as well as thrombotic conditions.

### Effect on cholinergic levels

Central cholinergic system dysfunction induces hippocampal neuronal loss, decreased choline acetyltransferase and acetyl cholinesterase activity, and decreased muscarinic and nicotinic receptor density. Resultant neuronal damage manifests with memory and learning disorders (Jiamou et al., 2020; Ying et al., 2022).

The phenolic compounds erulic acid and alkaloid ligustrazine found in Ligustici are not metabolized and freely permeate the blood-brain barrier to reach the hippocampus (Mancuso and Santangelo, 2014; Koh, 2015; Qian and Zengchun, 2019). Like acetyl cholinesterase inhibitors, these compounds increase the amount of acetylcholine and exert neuroprotective effects (Yunfeng and Huixia, 2011). Gastrodin extract treatment was reported to shorten mouse escape time and platform search distance, as well as increase the amount of cerebral acetylcholine and alleviate dysmnesia induced by scopolamine (Chunni et al., 2014). Ginseng (Ying et al., 2014; Md et al., 2018), and in particular ginsenosides, play important roles in protecting against oxidative stress and regulating cholinergic signaling. Most notably, ginsenoside Rb1 was reported to increase hippocampal antioxidant levels (Rui-xi et al., 2015; Zhu et al., 2019). Quercetin extracted from Euonymus alatus was similarly reported to increase acetylcholinesterase levels (Rui-xi et al., 2015; Sharma et al., 2021; Poonam et al., 2022).

### Effect on levels of inflammatory mediators

Neuroinflammation promotes VaD pathogenesis and imbalances in proinflammatory and antinflammatory factor secretion is relevant to involved pathomechanisms. A number of inflammatory markers have attracted attention as potential novel biomarkers due to changes in their levels early in VaD pathology. The inflammatory cascade is activated (Roux et al., 2001; Wyss-Coray and Mucke, 2002) by TNF-α, a monokine mainly produced by monocytes and macrophages. Belkhelfa et al. (2018) reported levels of TNF-α and IL-1β in the VaD hippocampus to be significantly higher as compared to those of control individuals. Their interaction with TLR-4, a receptor distributed mainly on the microglial cell surface, results in proinflammatory factor activation and neurodegeneration. Importantly, SMYZG decreases levels of IL-1β and TNF-α in VaD model rats.

Ferulic acid, a component of Ligustici, likely functions via ERK signaling (Guifang et al., 2021; Yuanyuan et al., 2021) to repress microglial activation and neuroinflammation. Ginsenosides (Bo et al., 2019) decrease levels of TLR3 and TRIF mRNA, resulting in activation of the TLR3/TRIF pathway and attenuation of inflammation in VaD rats. Gastrodia elata (Huan et al., 2017) ameliorates oxidative stress and inflammation and promotes humoral immunity, while quercetin (Poonam et al., 2022) decreases IL-6, IL-10, TNF-α, and acetylcholinesterase levels in VaD rats, attenuating endothelial dysfunction associated with hypertension.

Studies based on 2-VO rats suggest (Sun et al., 2020; Lijuan et al., 2022) that the level of 1L-1β and TNF-α increased in the model group, while decreased in SMYZ groups and had dose-effect relationship. Indicated that SMYZ can improve the learning and memory ability of rats with vascular cognitive disorder by inhibiting inflammatory response and improving oxidative stress state.

### Effect on antioxidative stress system

The oxidative stress response increases the level of reactive oxygen species depletes antioxidative compound stores and negatively impacts neuronal survival (Agdeppa et al., 2003), thus damaging synaptic activity in the area of involvement. Alterations in subsequent neurotransmission result in cognitive impairment (Tönnies and Trushina, 2017).

Ginsenosides are known to exhibit antioxidative properties (Dongmin et al., 2020); the ginsenoside Rg1 was reported to reduce oxidative injury and attenuate cognitive impairment (Chen et al., 2018). In addition, this compound was reported to exert positive effects in the setting of cerebral ischemia-reperfusion injury (Chu et al., 2019). Ferulic acid is neuroprotective primarily via mechanisms that decrease intracellular oxidative stress (Jianliang et al., 2015; Ling et al., 2019). Both LT and aqueous extract of Ligustici decrease malondialdehyde (MDA) levels in hypoxic neurons and increase superoxide dismutase activity. Gastrodia extract passes through the blood-brain barrier and exerts effects directly on brain tissue to normalize hemangiectasis and increase blood flow to the ischemic area (Chunyan et al., 2009), thereby lessening neuronal injury. This extract also reduces cellular calcium overload and decreases toxic effects of excitatory amino acids to produce an anti-apoptotic effect (Yunlin, 2006; Qihai et al., 2011; Aili, 2018). Debin and Shaofen (2006) reported that flavonoids prevent MDA generation via decreasing levels of H_2_O_2;_ quercetin (Wei Si-Can and Tian-lai, 2020) was found to decrease MDA levels and increase superoxide dismutase activity.

Studies based on 2-VO rats suggested (Yu Cao, 2019; Sun et al., 2020) that the level of SOD, GSH, and GSH-Px decreased significantly, the level of MDA increased in the model group. While the level of SOD, GSH, and GSH-Px increased, the level of MDA decreased in SMYZ groups. Indicated that SMYZ can depress activation and proliferation of glial cells in hippocampal CA1 region, and improve mitochondrial ultrastructure.

### Effect on cerebral white matter myelination

Cerebral white matter lesions are the most common pathological marker of VaD (Prins and Scheltens, 2015; Alber et al., 2019), manifesting in myelin discontinuity (Hill and Grutzendler, 2019). Levels of myelin basic protein, a membrane protein found on the serosal myelin surface, is significantly related to the severity of myelinoclasis. It also serves as an important indicator of demyelinating disease severity and remyelination (Choi et al., 2016; Nasrabady et al., 2018). White matter lesions occur due to a number of etiologies such as hypoxia, oxidative stress and the resultant inflammatory response, blood-brain barrier permeability and neurovascular unit disorder (Li Zehui, 2020).

The ginsenoside Rb1 was previously reported to improve symptoms of neurologic impairment by increasing lacunar infarct density in areas of infarction via the promotion of angiogenesis-related factor expression, such as VEGF and Ang-2 (Xiao et al., 2019). This compound was also reported to promote cortical neuronal stem cell proliferation and differentiation toward neuroglia–like cells (Wang et al., 2009). *In vitro* studies of LT (Qin et al., 2018) revealed that phthalide compounds such as ligustilide and senkyunolide A facilitate the penetration by certain medications of the blood-brain barrier. Ligustilide protects damaged nerves by attenuating cerebral ischemia-reperfusion injury via antioxidant and anti-apoptotic activity (Peng et al., 2007; Li et al., 2017). Studies on models of corneal endothelial injury (Guofeng et al., 2012) revealed that LT protects the endothelia via amelioration of cell damage by inhibiting expression of COX-2 and NF-κB proteins, as well as decreasing MAPK phosphorylation. Phenolic compounds found in Gastrodia were reported to exert neuroprotective effects by decreasing hippocampal NO content and NOS activity, reducing nNOS and iNOS expression and promoting eNOS expression (Xiaohua et al., 2011; Table 1).

**TABLE 1:** Pharmacologic effects of active ingredients composing Shen Ma Yi Zhi granule (SMYZG).

Article	Plant name	Active ingredient	Chemical structure type	Pharmacological action index	Signaling pathways	Effects
Koh (2015) and Qian and Zengchun (2019)	Ligustici	Ferulic acid	Phenolic compounds C10H10O4	MDA,SOD, GSH, IL-6, IL-10, TNF-α, AchE	(1) Nrf2/HO-1 pathway	(1) Anti-oxidative stress
Mancuso and Santangelo (2014)		Ligustrazine (LT)		AchE	(1) Nrf2/HO-1 pathway	(1) Anti-oxidative stress
Xiao et al. (2019)	Ginseng	Ginsenoside Rg1		TLR3 protein, TLR3 mRNA, TRIF protein, and TRIF mRNA	(1) Nrf2/HO-1 pathway (2) VEGF/Flk-1/P38MAPK signaling	(1) Anti-oxidative stress (2) Repair neure injury
		Ginsenoside Rb1		B-Secretases, Presenilin-1 (PS-1)	(1) AMPK/PPARα/PGC-1α/UCP2 pathway (2) Nrf2/HO-1 pathway (3) HIF-1/VEGF/Notch signaling (4) Pericyte signal	(1) Repair neure injury and mitochondrial injury inhippocampus (2) Suppress inflammatory response (3) Increase microvessel density
Chunyan et al. (2009), Xiaohua et al. (2011), and Wei et al. (2019)	Gastrodia elata	Gastrodin	Phenolic compounds	NO, iNOS, AchE, and VEGF	(1) Nrf2/HO-1 pathway (2) Pericyte signal (3) AMPK/PPARα/PGC-1α/UCP2 pathway (4) VEGF/Flk-1/P38MAPK signaling	(1) Anti-oxidative stress, suppress inflammatory response and anti-apoptosis (2) Maintain and recover physiological function of cerebrovascular (3) Increase oxygen utilization rate of brain and improve energy metabolism (4) Anti-oxidative stress, anti-apoptosis, suppress calcium overload and decrease excitatory toxicity
Wei Si-Can and Tian-lai (2020)	Euonymus alatus	Quercetin		MDA, SOD, IL-6, IL-10, TNF-α, and AchE	(1) Nrf2/HO-1 pathway	(1) Improve energy metabolism and improve blood rheology (2) Anti-oxidative stress
Debin and Shaofen (2006)		Flavonoids		MDA and SOD	(1) Nrf2/HO-1 pathway	(1) Scavenging free radical (2) Anti-oxidative stress

### Signaling pathways relevant to vascular dementia treatment by Shen Ma Yi Zhi granule

Mechanistic studies of VaD have investigated apolipoprotein, tau and lipid metabolism, as well as immune and oxidative stress responses. Although amyloid-ß is currently considered to be the initiator of pathological changes in the setting of Alzheimer’s disease, genetic susceptibility factors mediating reactions to amyloid-ß such as metabolic processes, immunity and lysosomal function are of vital importance in the pathogenesis of VaD and Alzheimer’s.

#### Effect on mitochondrial metabolism

Chronic cerebral hypoperfusion refers to cerebral low-flow ischemia and hypoxic conditions due to angiostenosis. Chronic cerebral hypoperfusion is itself a risk factor for cognitive impairment and patients suffering cognitive decline commonly suffer significant cerebral hypoperfusion (Cao et al., 2016; Kalaria, 2016; Kazumata et al., 2019), and vice versa (Jessica et al., 2017).

Gastrodin was reported to increase neuronal oxygen metabolism, neuronal levels of ATP, promote neuronal glucose uptake and utilization, enhance memory and decrease lactate generation in the setting of ischemia (Meikang et al., 2010). The ginsenoside Re was reported to regenerate damaged neurons and improve cognition in rats via normalization of mitochondrial physiology (Wang et al., 2009).

**FIGURE 5:**
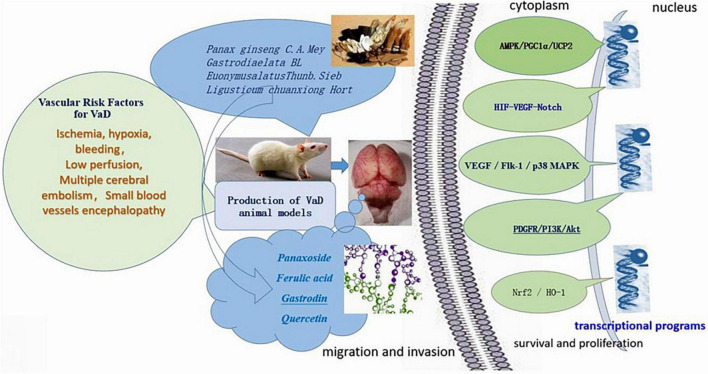
Signaling pathways of Shen Ma Yi Zhi granule (SMYZG) in the context of vascular dementia (VaD) treatment. Sources for the images within this figure: 699pic.com and Chinese Pharmacopoeia.

Shen Ma Yi Zhi granule improves neuronal mitochondrial metabolism in 2-VO rats via the AMPK/PPARα/PGC-1α/UCP2 pathway (Sun et al., 2021b). Levels of AMPK, PPARα, PGC-1α, and ATP5A protein and mRNA were reported to be increased in the setting of SMYZG treatment, while levels of UCP2 gene expression were reported to be decreased (Figure 5).

#### Effect on anti-oxidative stress and ant-inflammatory signaling

Oxidative stress and inflammation directly damage cells (Beydoun et al., 2022). Bioactive products in the setting of oxidative stress initiate the inflammatory response (Wang et al., 2007) and are known to significantly aggravate cerebral injury. Astrocytes supply neuronal energy (Elkabes et al., 1996; Nakajima et al., 2001; Changhai and Rui, 2007; Gardiner et al., 2009), improve neuronal function, and promote memory generation and consolidation via secretion of BNDF (Allen et al., 2013). The hippocampal inflammatory response is the main pathologic characteristic of VaD (Stefaniak and O’Brien, 2016; Price et al., 2018). Nrf2 and HO-1, key signal molecules of the Nrf2/HO-1 pathway, exert anti-oxidative and ant-inflammatory effects (Wang et al., 2015).

The ginsenoside Rb1 improves learning and memory via attenuation of the hippocampal inflammatory response in a rat model of cerebral ischemia-reperfusion (Wang et al., 2009). Senkyunolide I reduces cerebral inflammation caused by oxidative stress and glucose deprivation as well as reoxygenation. Flavonoids and total steroids found in Euonymus alatus were found to possess signaling capabilities relevant to maintenance of oxygen free radical levels (Debin and Shaofen, 2006). Studies on VaD rats revealed that quercetin (Wei Si-Can and Tian-lai, 2020) administration resulted in increased neuronal lactate dehydrogenase activity and ATP levels as well as inhibition of cerebral NOS activity. Neuronal NO and lactate dehydrogenase levels as well as serum ET-1 levels were reported to be decreased, while serum levels of calcitonin gene-related peptide increased, thus promoting physiological neuronal metabolism and functioning. SMYZG (Kun et al., 2020; Wei Si-Can and Tian-lai, 2020) improves cognitive function and exerts neuroprotective effects by promoting the proliferation of reactive astrocyte-type cells via upregulation of the Nrf2/HO1 pathway.

#### Effect on the neurovascular unit

The neurovascular unit consists of the blood-brain barrier, neurons and glial cells including astrocytes, oligodendrocytes microglia. The neurovascular unit is a fairly novel concept that has attracted interest for consideration in research of stroke treatment (Muoio et al., 2014). The neurovascular unit together with pericytes as well as the corneal epithelial cells are critically important in the pathogenesis of ischemic cerebrovascular and hemorrhagic cerebral vascular diseases (Ozgur et al., 2017).

##### Effect on pericyte signal

Pericytes play critical roles in areas of lacunar infarction (Mark, 2009). Pericytes function to regulate blood-brain barrier permeability, clearing cellular components (Ferland-McCollough et al., 2017), maintaining homeostasis of cerebral vascular and protecting the central nervous system.

Surgeries (Sun et al., 2021a) can cause vascular injury and angiogenic instability via downregulation of Ang1 and PDGFR-β protein expression in corneal epithelial cells of brains among MID rats. SMYZG was reported to promote Ang1 and Ang1 mRNA expression in pericytes, increase neuronal PDGFR-β and PDGFR-β mRNA levels, and increase NG2 and NG2 mRNA levels. These results underscore that SMYZG protects pericytes and improves neurovascular homeostasis.

##### Effect on angiogenesis signaling

Vascular endothelial growth factor, astrocytes, Ang-1, Notch, and pericytes were found to synergistically interact to stimulate regeneration and maturation of blood vessels (Li, 2017; Kun et al., 2020). The hypoxia inducible factor-1 (HIF-1)-VEGF and Notch pathways are two main signaling pathways in angiogenesis. HIF-1 is one transcription factor relevant to intracellular oxygen homeostasis that is evoked by hypoxia. VEGF, another neuroprotective factor increased in neuronal tissue in the setting of cerebrovascular trauma, decreases blood-brain barrier permeability and promotes angiogenesis (Lian Jin et al., 2011). The Notch gene plays a key role in HIF-1/VEGF/Notch signaling and thus promotes blood vessel regeneration (Luttun et al., 2002; Yin, 2016).

##### Effect on endothelial cells and synaptic plasticity in the context of lacunar infarction

Endothelial cells of the blood-brain barrier are important early in the pathogenesis of VaD. Injury of proteins involved in neural synaptic plasticity is also important in cognitive impairment. Endothelial cells regulate neuronal activation via secretion of compounds such as VEGF, BDNF, and NO. Similarly, endothelial cells regulate synaptic plasticity via VEGF/Flk-1/p8 MAPK signaling.

Studies (Lian Jin et al., 2011; Li, 2017; Xuemei et al., 2019) of VaD rat models reported that SMYZG promotes expression of P38MAPK, NMDAR1, PSD-95, VEGF, and FLK-1 mRNA and protein in rat neuronal tissue, promotes expression of choline acetyltransferase, inhibits MMP9 expression. SMYZG was reported to improve cognition via inhibition of neuroglial cell activation and proliferation in the CA1 region. SMYZG also improves endothelial cellular function, activates VEGF/Flk-1/P38MAPK signaling and improves synaptic plasticity (Table 2).

**TABLE 2:** Likely signaling pathways of Shen Ma Yi Zhi granule (SMYZG).

Articles	Signal pathways	Animal models	Target proteins	Regulate trends	Mechanism
Sun et al. (2021b)	AMPK/PGC-1α/UCP2/ATP5A	2-VO	AMPK, PPARα, PGC-1α, and ATP5A	Up	Mitochondrial related proteins, mitochondrial energy metabolism
			UCP2	Down	
Sun et al. (2021a)	Pericytes, Ang1, PDGFR-β protein	MID	Ang1 and PDGFR-β	Up	Pericytes, pathogenesis of neovascularization
Kun et al. (2020)	HIF/VEGF Notch	MID	HIF1α, VEGF, VEGF mRNA, and notch	Up	Pathogenesis of neovasculariza
Xuemei et al. (2019) and Yu et al. (2019)	Nrf2/HO-1	MID	Nrf2mRNA, HO-1mRNA	Up	Antioxidant stress response
Li (2017)	VEGF/Flk-1/P38MAPK	2-VO	P38MARK,NMDAR1, PSD-95, VEGF, FLK-1 mRNA, and proteins	Up	Synaptic plasticity improving via vascular endothelium

## Conclusion and perspectives

Vascular dementia (Gorelick et al., 2011) is a common, highly heterogeneous condition that manifests due to stroke, neurovascular trauma or cerebrovascular disease. The four major subtypes of VaD that can be further subclassified include MID, post-stroke dementia, subcortical ischemic vascular dementia and mixed dementia (Skrobot et al., 2016a,b, 2018; Hao et al., 2021). VaD is the second commonest dementia after Alzheimer’s, and although Alzheimer’s is characterized by episodic disturbances in memory, both conditions are clinically similar and challenging to distinguish (Laukka et al., 2004). Interestingly, VaD and Alzheimer’s were reported to commonly coexist in patients suffering cognitive impairment (Schneider et al., 2005; Sonnen et al., 2007; Launer et al., 2008; Wu, 2019). And end-stage VaD patients share the similar pathological basis with AD patients (Wu, 2019). As interest in VaD research increases, particular focus on treatment improvement, pathological mechanism elucidation and conduction of prospective drug development studies is warranted (Cognitive Impairment Committee NBCM, 2019; Hao et al., 2021).

**FIGURE 6:**
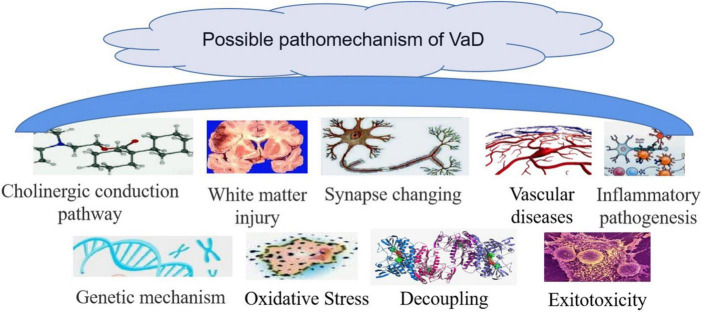
Possible pathomechanism of vascular dementia (VaD). Sources for the images within this figure: 699pic.com and Chinese Pharmacopoeia.

Chronic cerebral hypoperfusion is the main etiological mechanism of VaD (Cao et al., 2016; Jessica et al., 2017; Kazumata et al., 2019), with half of VaD patients suffering subcortical white matter infarction (Kalaria, 2016). However, white matter lesions are considered to also be an etiology for chronic cerebral hypoperfusion and a pathologic characteristic common in both Alzheimer’s and VaD (Figure 6).

The concept of the neurovascular unit underscores the relationship between VaD and other cerebrovascular diseases. Investigation of interactions among neurons, glial cells and the cerebral vasculature should be encouraged from a holistic perspective. A variety of active compounds compose SMYZG such as ferulic acid, LT, ginsenoside Rg1, ginsenoside Rb1, gastrodin, and quercetin, as well as a number of flavonoids. Treatment targets have been reported to include dementia-associated proteins (e.g., beta-secretase, PS-1), apoptosis-associated factors (e.g., TLR3, TRIF), vascular endothelium- -associated factors (i.e., VEGF), components of cholinergic signaling (e.g., acetylcholinesterase, choline), inflammatory factors (e.g., IL-6, IL-10, NF-α) and factors important in oxidative stress (e.g., MDA, SOD, GSH, NO, iNOS). To further clarify effects of SMYZG in the clinical setting, multi-center randomized controlled trials with large sample sizes are required. Clinical monitoring of SMYZG blood levels as well as investigation of pharmacokinetic properties, are also warranted.

This article reviewed previously published data from studies using different animal models relevant to the pharmacological mechanism, pharmacokinetics and clinical applications of SMYZG, as well as the pathophysiological characteristics of VaD. Important processes ameliorated by SMYZG treatment include neuronal oxidative stress, cerebral white matter demyelination, pericyte function, neuronal mitochondrial metabolism, cerebral endothelial function and neuroinflammation. Relevant signaling pathways include the AMPK/PPARα/PGC-1α/UCP2, Nrf2/HO-1, HIF-1/VEGF/Notch, and VEGF/Flk-1/p8 MAPK pathways.

In this manuscript, we collected around 10 years reports for SMYZ of VaD prevention and treatment. For future applications, more approaches need to be designed in further studies along with the development of proteomics, metabonomics, transcriptomics, network pharmacology, and interdisciplinary research. To identify active ingredients based on the sense of system biology. To evaluate pharmaceutical and pharmacological effects of active ingredients based on metabonomics. To research complex components of herbal medicines by establishing combination-activity relationship (CAR) according to the relevance of herbal combination and bioactivity based on systems modeling (Liu et al., 2016; Liu, 2017). Various metabolites will be produced during the whole process after oral administration of SMYZ to form pharmaco-metabonomics (Xiao, 2014; National Medical Products Administration, 2020). We shall test active components in plasma, prototype components, gut bacteria and metabolites with approaches like nuclear magnetic resonance (NMR), gas chromatography-mass spectrometry (GC-MC) and ultra performance liquid chromatography-tandem mass spectrometry (UPLC-MS/MS). This methods are conducted by modern equipments that are characterized by high resolution, high-throughput and high sensitivity. Using of all above mentioned technologies is consistent with concept of holism of Traditional Chinese Medicine (TCM).

Chinese medicines are complicated chemical systems based on systematic chemical separation, biological expression, separation and preparation of herb components. For further study, our research group will put more efforts on figuring out the compatibility regularity and integrating multi-components. The basic pharmacology, mechanism of action and the relationship between active components and clinical efficacy of the drug must be determined including toxicologic effects. Upgrade the preparation technique and make new traditional Chinese medicines with controllable quality, low toxic and clearly mechanisms.

## Author contributions

J-GL and HL conceived the topic and helped to draft the manuscript. J-GL and S-RC wrote the manuscript together. M-XL, D-DS, and L-JZ participated in the research. All authors contributed to the article and approved the submitted version.
